# Trophic transfer and bioaccumulation of nanoplastics in *Coryphaena hippurus* (mahi-mahi) and effect of depuration

**DOI:** 10.1371/journal.pone.0314191

**Published:** 2024-11-21

**Authors:** Preyojon Dey, Terence M. Bradley, Alicia Boymelgreen

**Affiliations:** 1 Department of Mechanical and Materials Engineering, Florida International University, Miami, Florida, United States of America; 2 Department of Fisheries, Animal and Veterinary Science, University of Rhode Island, Kingston, Rhode Island, United States of America; Jiangsu University, CHINA

## Abstract

Ocean plastic pollution is a global concern, exacerbated by the distinctive physiochemical characteristics of nanoplastics (NPs), making it crucial to study the impacts on marine animals, particularly fish, given their ecological and economic importance. Both trophic transfer and waterborne exposure are potential modes of NP entry into seafood for human consumption Although the majority of studies have focused on in-vitro impacts of NP exposure in fish, in-vivo methods can offer a more holistic understanding of these impacts. This study investigates polystyrene NP transfer to *Coryphaena hippurus* (mahi-mahi) larvae, a widely consumed fish and significant marine predator, during the early life stage. *Brachionus plicatilis* (rotifers) were exposed to NPs, and subsequently fed to *C*. *hippurus* larvae, with exposure duration ranging from 24 to 96 h. Significant NP transfer was observed via the food chain, varying with exposure duration. A depuration study over 72 h, simulating intermittent NP exposure, revealed substantial NP excretion but also notable retention in the larvae. Biodistribution analysis indicated that most NPs accumulated in the gut, with a significant portion remaining post-depuration and some translocating to other body areas containing vital organs like the heart, liver, and gall bladder. Despite no significant effects on body length and eye diameter during this short study period, histopathological analysis revealed intestinal tissue damage in the larvae. Overall, this study provides valuable insight into the trophic transfer of NPs in marine food webs, emphasizing the need for further research on ecological impacts and highlighting the importance of addressing NP contamination to protect marine ecosystems and food safety.

## 1. Introduction

Plastics are extensively utilized throughout a wide range of applications owing to their cost-effectiveness, lightweight nature, and enhanced properties. However, a mere 9% of the plastics generated undergo recycling, leading to a significant proportion of plastic waste being disposed of [1]. Approximately 3% of total plastic produced is deposited into the ocean by various pathways such as rivers, runoffs, and direct discharges [2], and has emerged as the predominant marine pollutant, comprising 60–80% of the total marine litter [3].

Prior to reaching the ocean, plastic waste undergoes degradation processes on land, resulting in a range of sizes from macro (5–100 mm) to nanoplastics (NPs) (<1 μm) [1]. The predominant form of marine plastic waste is macroplastics, accounting for approximately 70–80% of the total [4]. These macroplastics also undergo degradation and fragmentation in the marine environment through several processes, including photochemical reactions, mechanical forces, hydrolysis, and biological activities, resulting in the formation of secondary microplastics (MPs) and NPs [4,5].

NPs pose a significant risk to marine organisms because of their smaller size and weight, as well as their increased surface area [6,7], which facilitates widespread dispersion [8,9], potential ingestion as food [10,11], and higher adsorption and faster leaching [12,13]. For example, it has been observed that direct exposure to NPs has resulted in adverse effects in marine animals such as embryotoxicity and abnormal gene expression in the sea urchin *Paracentrotus lividus* [14]; inhibition in hatching [15], feeding [16], and swimming [15,16], molting [16] as well as mortality [15] in *Artemia*; rapid destabilization of lysosomes, production of reactive oxygen species (ROS) and nitric oxide, and inhibition of phagocytosis in the marine bivalve *Mytilus galloprovincialis* [16], etc.

Most studies on NP toxicity are limited to exposure via the waterborne route, with information on trophic transfer—an important pathway for NP exposure—remaining limited despite its potential for significant impacts. For example, NPs have been observed to be transferred to the upper trophic level via the food chain, such as the food chain consisting of NPs, *D*. *magna*, and *Carassius carassius* [17,18], and to have caused disturbances in feeding, shoaling, and metabolism in upper trophic animals [17]. Given that fish constitutes a significant part of the marine ecosystem [19,20] and a major source of animal protein for humans [21], it is crucial to understand how NPs affect marine fish species, particularly at their vulnerable early life stages. In this context, marine fish are hypothesized to take up and retain significant amounts of NPs through the food chain, with uptake and retention influenced by the duration of exposure. This NP retention may not be fully reversible, suggesting that NPs could persist within fish even after exposure ceases, accumulating primarily in the gut but potentially translocating to other organs, resulting in tissue damage, abnormalities, and impaired growth. Although in-vivo studies offer a more realistic understanding, most existing fish nanotoxicity studies are in-vitro [22,23], with the few in-vivo studies primarily focusing on *Danio rerio* [24–26], a freshwater species. The behavior of NPs differs in freshwater and saltwater [27] and is expected to influence their impacts on marine species [28]. Understanding NP retention in fish is crucial, as they can potentially transfer to humans through the food chain. The uptake of plastic or plastic additives and derivatives is associated with various metabolic and functional disorders [29], carcinogenicity [30], neurotoxicity [31,32], obesity [33], as well as hemolysis and eryptosis in erythrocytes [34] in humans.

To fill this research gap and evaluate our hypotheses, this study investigates the ingestion, retention, and distribution of NPs in *Coryphaena hippurus* (mahi-mahi or dolphinfish) larvae exposed to NPs through the food chain for varying durations. It also assesses the effects of NPs on normal growth and potential abnormalities by measuring length and eye diameter, two parameters used in previous studies for similar purposes [35–37]. Additionally, histopathological analysis is performed to investigate NP trophic transfer-related tissue damage in the intestines. *C*. *hippurus* is extensively harvested and utilized as a food source for human consumption. Investigation of the potential impacts of NPs on this economically and ecologically significant species is important, with a focus on determining the effects of NPs, whether NPs are retained, and the potential implications for human food safety. While there have been studies investigating the effects of environmental disruptions, such as oil spills [38–41] and abiotic environmental factors [42,43], on *C*. *hippurus*, there is a lack of research on foodborne exposure to NPs. This route might transfer higher quantities of NPs compared to the waterborne route [44], highlighting the need to investigate its implications for this important species. The lower trophic organism employed in this study was the marine zooplankton species *Brachionus plicatilis* (rotifer). *B*. *plicatilis* has frequently been utilized as a model organism in ecotoxicological investigations owing to its availability and cultivation, as well as its rapid growth rate [45–48]. In this study, polystyrene (PS) NPs were used as a model due to their widespread use in various applications [49,50] and their prevalence in marine litter [51–53]. Polystyrene NPs are also commonly employed in ecotoxicological studies [15,17,18,54–56]. Following NP exposure, *C*. *hippurus* larvae were subjected to depuration for varying durations to assess the reversibility of NP retention in the absence of continued NP exposure.

## 2. Materials and methods

### 2.1 Nanoplastic characterization

**Fig 1:**
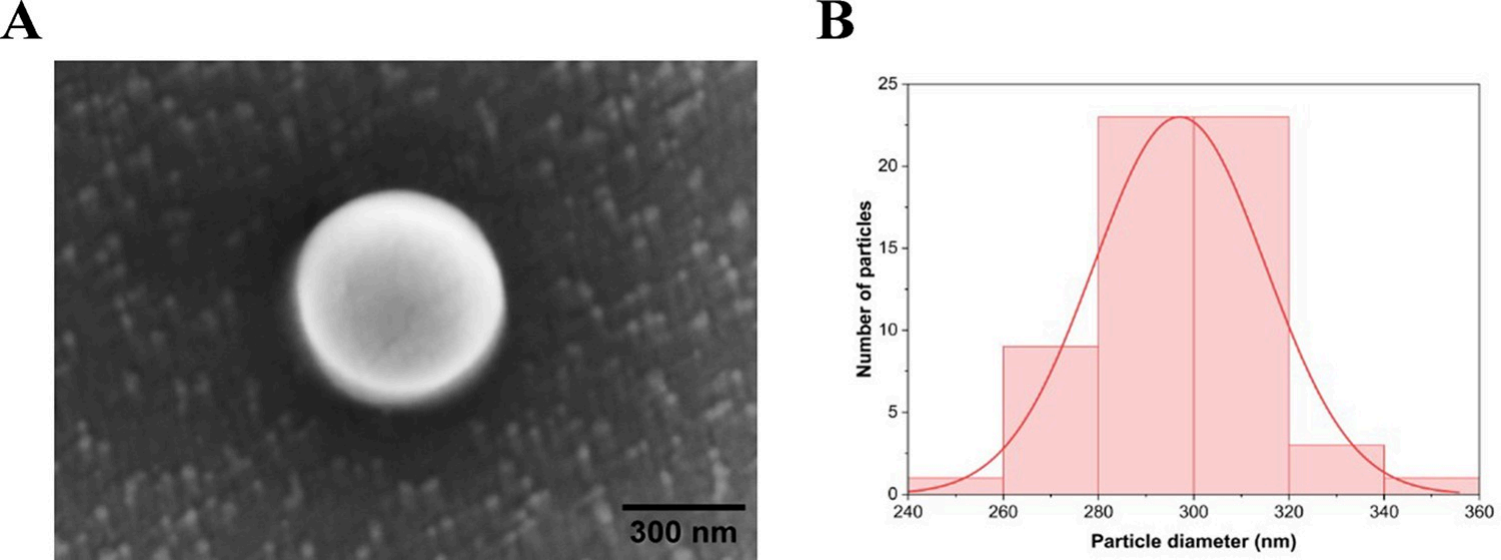
Nanoplastic shape & size. A) Scanning electron microscopy (SEM) images reveal the shape of the polystyrene nanoplastics used in this study (scale bar = 300 nm). B) Size (diameter) distribution of the nanoplastics from SEM image.

PS NPs containing tetramethylrhodamine (TRITC) fluorescent dye with a nominal size of 300 nm dissolved in an aqueous solution at a concentration of 1% solids (w/v) were procured from Thermo Scientific Chemicals (Fluoro-Max, cat. no. R300). Although the size of NPs has been considered less than 100 nm elsewhere [57,58], in many studies, plastics less than 1 μm are considered NPs [59–61], and the same was considered for this study. Furthermore, utilizing relatively larger NP sizes facilitates improved imaging of NP uptake and distribution solely through fluorescence microscopy, as discussed later in section 2.3. Before scanning electron microscopy (SEM) imaging (JEOL FS-100), as-received NP solutions were drop cast onto a double-sided carbon tape affixed to a glass coverslip and air dried overnight. SEM image revealed that PS NPs were spherical in shape (Fig 1A). SEM image analysis shows a size (diameter) distribution of 297.04 ± 18.03 nm (mean ± SD) for the NPs (Fig 1B). Dynamic light scattering (DLS) (Nano ZS, Malvern instruments) results show a Z-average size of 287.24 ± 1.82 nm, a polydispersity index (PDI) of 0.18 ± 0.04, and a Zeta potential of -0.82 ± 0.94 mV, respectively (mean ± SD). PS NPs were suspended in filtered seawater using a vortex mixer (Vortex Genie 2, Scientific Industries) to make NP suspensions with concentrations of 10 mg/L.

### 2.2 Nanoplastic exposure

Approximately 150,000 *B*. *plicatilis* was transferred to a beaker (1 L) containing NP suspension and held for 3 h. These NP-exposed (NPE) *B*. *plicatilis* were transferred at a concentration of 30 rotifers/mL, to a container with 100 *C*. *hippurus* larvae. At least five *B*. *plicatilis* larvae were extracted from the container at each exposure time point (24, 48, 72, and 96 h), euthanized, and preserved in a 70% ethanol solution in preparation for imaging. Additionally, *C*. *hippurus* larvae exposed to NP-containing rotifers for 24 and 48 h were collected, transferred to a container containing *B*. *plicatilis*, which did not receive any NP exposure (non-NPE), for depuration purposes for 24, 48, and 72 h. Following depuration, the larvae were euthanized and collected in a 70% ethanol solution, a method commonly used in studies for preserving aquatic species [62]. The water in the depuration container was changed daily. We note that larvae exposed to NPs through dietary intake for 72 and 96 h did not survive thereafter, potentially due to prolonged NP exposure [63], thereby precluding the investigation of depuration effects on these groups. Additionally, variations in NP concentration and size may influence the survival outcomes of these test organisms [64], warranting further investigation to delineate these impacts. The control group of *C*. *hippurus* was only exposed to non-NPE rotifers and collected at the previously mentioned time points. Tables 1 and 2 summarize the study parameters and sample collection points, respectively.

**Table 1:** Study parameters.

NP concentration for *B*. *plicatilis*	NP exposure duration for *B*. *plicatilis*	*C*. *hippurus* larvae concentration	*B*. *plicatilis* concentration fed to *C*. *hippurus* larvae
10 mg/L	3 h	100 larvae/L	30 rotifers/mL (approximately 300 rotifers per larva)

**Table 2:** Sample collection time points.

Exposure duration (h)	Depuration duration (h)	Sample collection status
24	24	Samples collected
	48	Samples collected
	72	Samples collected
48	24	Samples collected
	48	Samples collected
	72	Samples collected
72		Sample collection was not possible due to larval mortality
96		Sample collection was not possible due to larval mortality

### 2.3 Image capture, processing, and histopathological analysis

**Fig 2:**
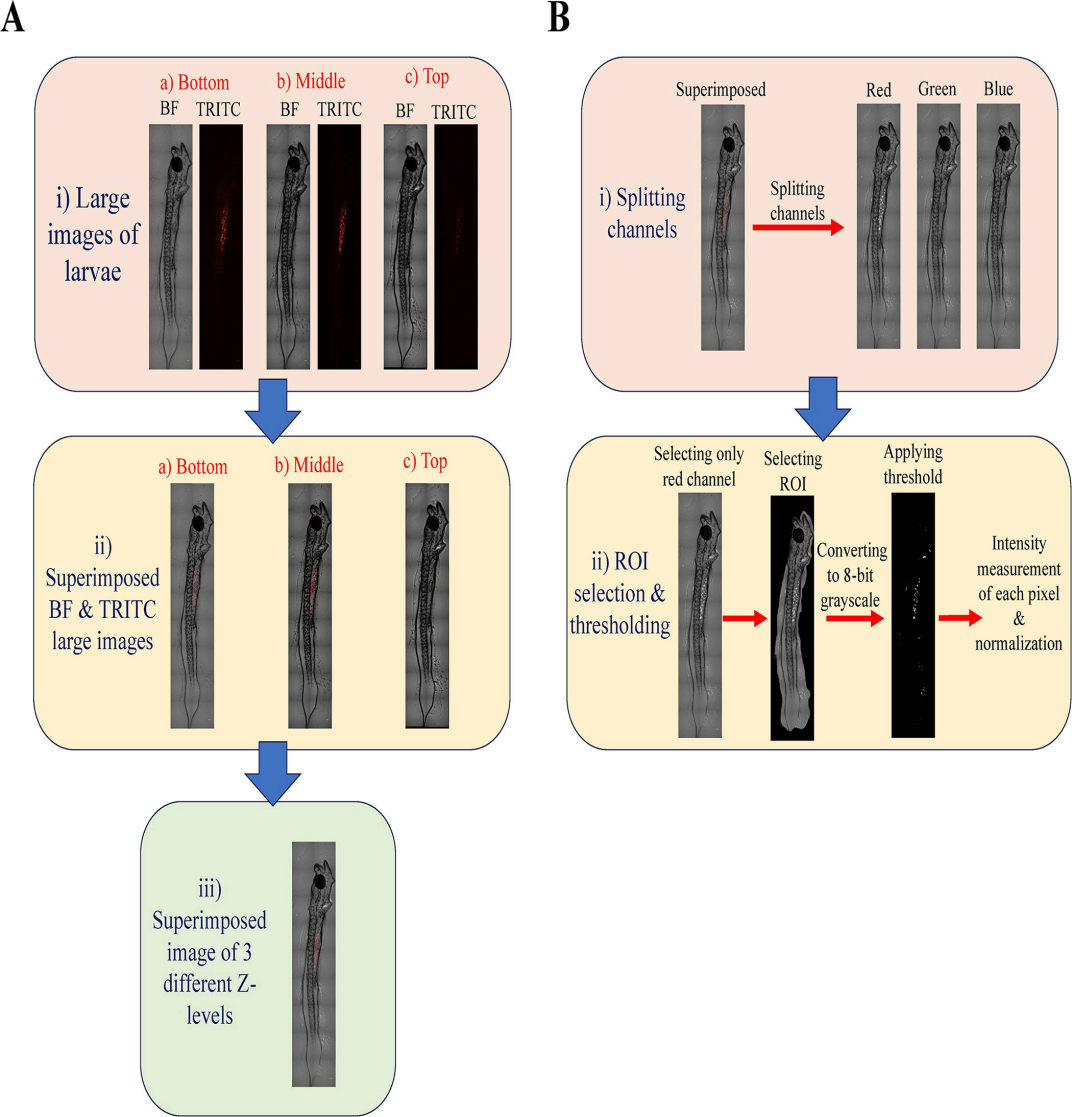
Image processing and analysis. (A) Sequential processing of larvae images- i) Acquisition of large images of larvae at various Z levels using different filters (BF: Bright-field image and TRITC: Fluorescence image), ii) Superimposition of BF and TRITC images of larvae at multiple Z levels, iii) Superimposition of images found from step A(ii). (B) Fluorescence intensity measurement- i) Splitting of the superimposed image from step A (iii) into red, green, and blue channels, ii) Isolation of the red channel image, identification of larvae as the region of interest (ROI), conversion into gray scale image, and application of a threshold and normalization via the control group to quantify fold change in fluorescence intensity.

The larvae samples were carefully placed within an antifade mounting media (Prolong^TM^ Glass Antifade Mountant, Invitrogen), sandwiched between a glass coverslip and slide. Subsequently, these samples were subjected to imaging using a spinning disc confocal fluorescence microscope (Nikon CSU-X1 mounted on Nikon Eclipse Ti2-E) under both fluorescence (TRITC) and bright-field filters. Images were captured at three distinct Z-levels (bottom, middle, and top) to comprehensively scan the entire depth of the larval samples [Fig 2A(i)]. Due to the microscope’s limited field of view at high magnification and the large size of the larvae sample, images of the entire sample were taken in segments and later stitched together automatically using the NIS-Elements program (large image option) [65]. Fluorescence and bright-field images from each Z-level were superimposed on each other and merged into a single composite image using ImageJ [Fig 2A(ii)]. Composite images from different Z-levels were stacked and processed into a single image [Fig 2A(iii)], which was subsequently divided into multiple channels (red, green, and blue) [Fig 2B(i)]. The red channel image was further processed by taking only larvae as the region of interest (ROI), converting it into an 8-bit grayscale image, and then applying Yen’s auto-thresholding method [66]. The fluorescence intensity of this image was quantified using ImageJ [67]. The quantified fluorescence intensity was then normalized against the control group to determine the fold change in fluorescence intensity [Fig 2B(ii)]. Additionally, the length and eye diameter of larvae samples were measured using ImageJ [37,68].

Histopathological analysis of the larvae intestine was conducted by embedding the samples in paraffin, sectioning at 6 μm, and staining with hematoxylin and eosin (H&E), following established protocols [69]. The glass slide-mounted stained sections were then imaged using bright-field microscopy (Axioscope 5, Zeiss).

### 2.4 Statistical analysis

Statistical analyses were performed with OriginPro (2024b, OriginLab). The data are shown as mean ± standard deviation. Statistical significance was assessed using a one-way analysis of variance (ANOVA), followed by Tukey’s post-hoc test to compare different treatments with the control group. The data were tested for normality using a normal quantile-quantile plot at 95% confidence before ANOVA testing. In all statistical analyses, the results were considered to be statistically significant when the p-value was below 0.05.

### 2.5 Ethical approval

All experimental protocols received approval from the Institutional Animal Care and Use Committees (IACUC) at Florida International University (FIU) and the University of Rhode Island (URI). This approval was granted through written consent (Protocol approval number: IACUC-20-052-CR02 for FIU and Protocol approval number: AN2021-021 for URI).

## 3. Results

### 3.1 Effect of different exposure periods on bioaccumulation

**Fig 3:**
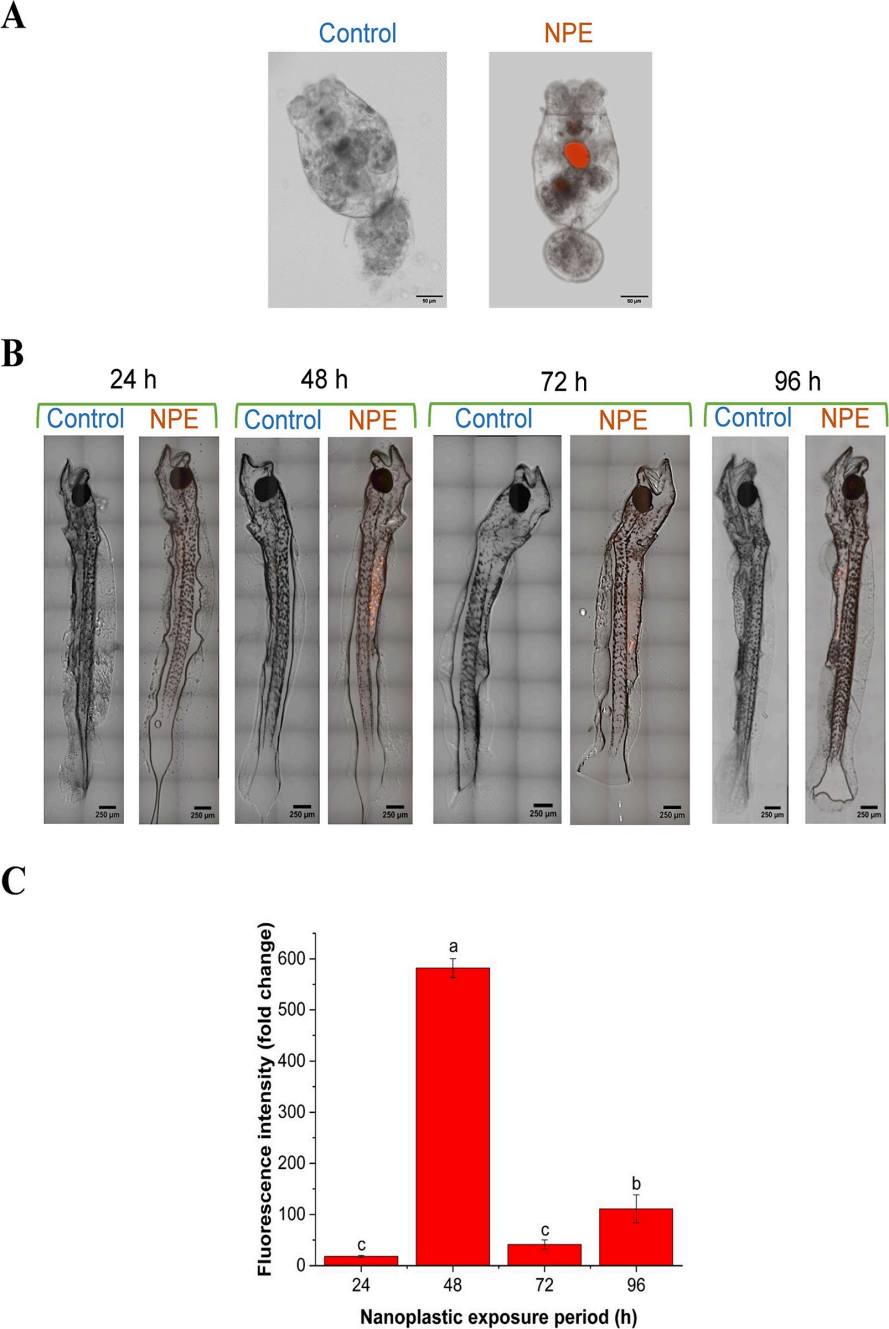
Bioaccumulation of NPs in *C*. *hippurus* through trophic transfer at different exposure periods. Superimposed bright field and fluorescence images demonstrate the presence of red fluorescent compared to the control in (A) *B*. *plicatilis* following waterborne exposure and (B) *C*. *hippurus* via trophic transfer after 3 h and various exposure periods, respectively. (C) Fluorescence intensity (fold change as compared to the control) of the red fluorescent nanoplastics accumulated in *C*. *hippurus* via trophic transfer.

Comparison of the superimposed bright field and fluorescence images (Fig 3A) reveals a significant uptake of red PS NPs by *B*. *plicatilis* within a brief exposure period of 3 h through waterborne exposure. Similarly, images of *C*. *hippurus* exposed to NPs via trophic transfer (Fig 3B) demonstrate significant uptake and bioaccumulation of NPs, which are absent in the control group. However, as seen by the red NP quantity, uptake and thus bioaccumulation varied with exposure durations. This observation is corroborated by fluorescence intensity (FI) measurements of *C*. *hippurus* (Fig 3C), which show a significant increase in FI fold change in the NPE group compared to the control group across all exposure periods, with an average fold change ranging from 18.11 to 581.95 depending on duration. A significant increase in FI with exposure was observed, rising from 24 to 48 h. Although FI decreased at 72 h compared to that at 48 h, it significantly increased again at 96 h.

### 3.2 Effect of different depuration periods on bioaccumulation

**Fig 4:**
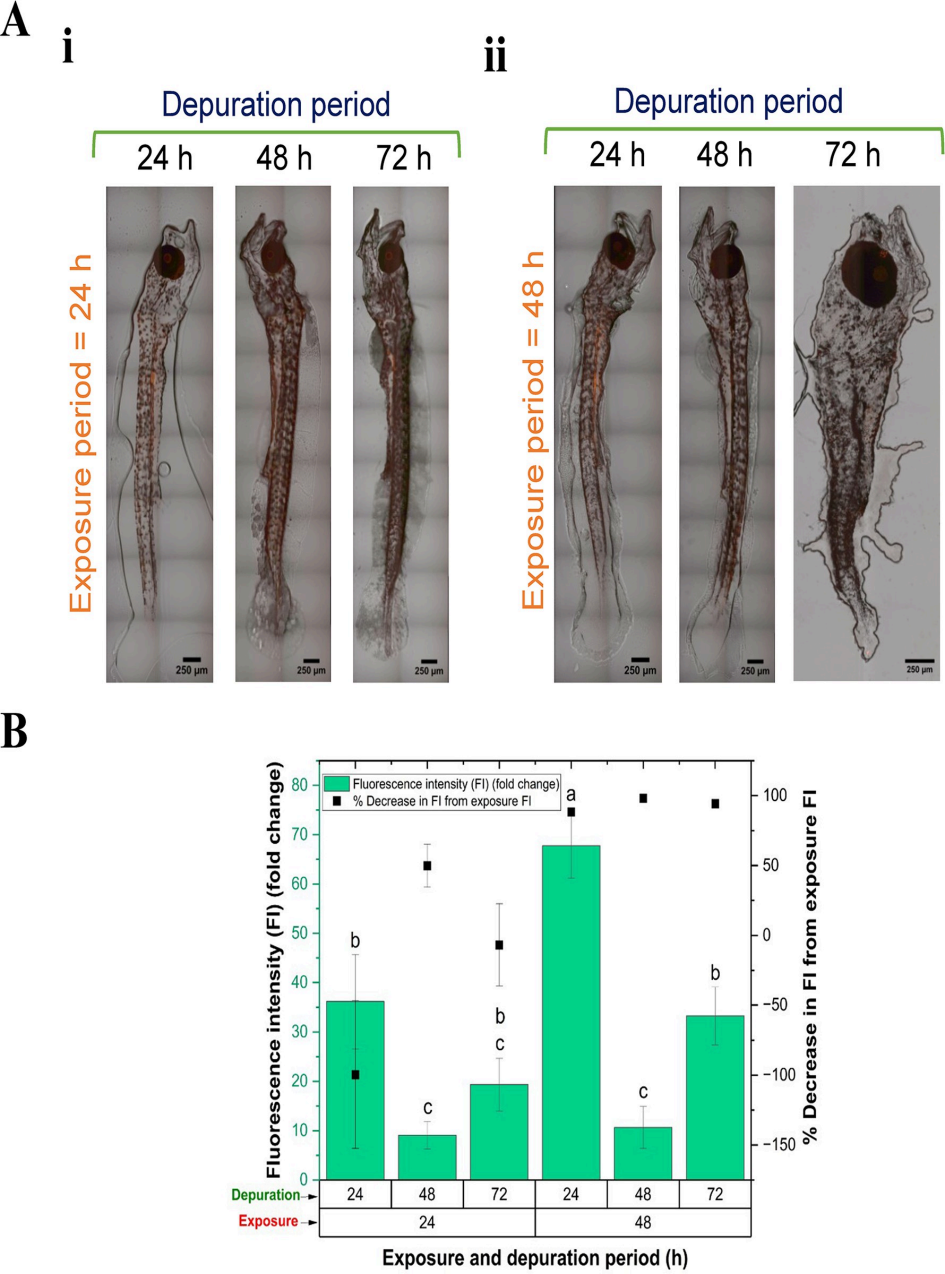
Effect of depuration period on the retention of NPs in *C*. *hippurus* following trophic transfer exposure for various durations. (A) Superimposed bright field and fluorescence images show the presence of red florescence nanoplastics in *C*. *hippurus* undergoing depuration. (B) Fluorescence intensity (FI) (fold change) of nanoplastics accumulated in *C*. *hippurus* after various depuration periods compared to the control, and % decrease in FI after depuration from corresponding post-exposure FI.

Fig 4 demonstrates the effect of different depuration periods on the bioaccumulation of NPs in *C*. *hippurus*, following varying NP exposure durations. Superimposed bright-field and fluorescence images (Fig 4A) highlight the presence of red fluorescent NPs in *C*. *hippurus*, absent in the control group, indicating retention of NPs transferred from *B*. *plicatilis* even after depuration. FI measurements (Fig 4B-left axis) confirm and quantify this retention, showing fluctuating NP levels across depuration periods. Fig 4B-right axis shows the % decrease in FI after a given depuration period compared to the FI at the corresponding exposure period. Notably, after 24 and 72 h of depuration following a 24-hour exposure, the FI increased unexpectedly as compared to that after 24 h of exposure, resulting in a negative % decrease in FI. Other conditions indicated significant FI decreases, with the highest bioaccumulation after 48 h of exposure showing an approximate 88% FI decrease after 24 h of depuration, further reducing to about 98% after 48 h.

### 3.3 Biodistribution of nanoplastics

**Fig 5:**
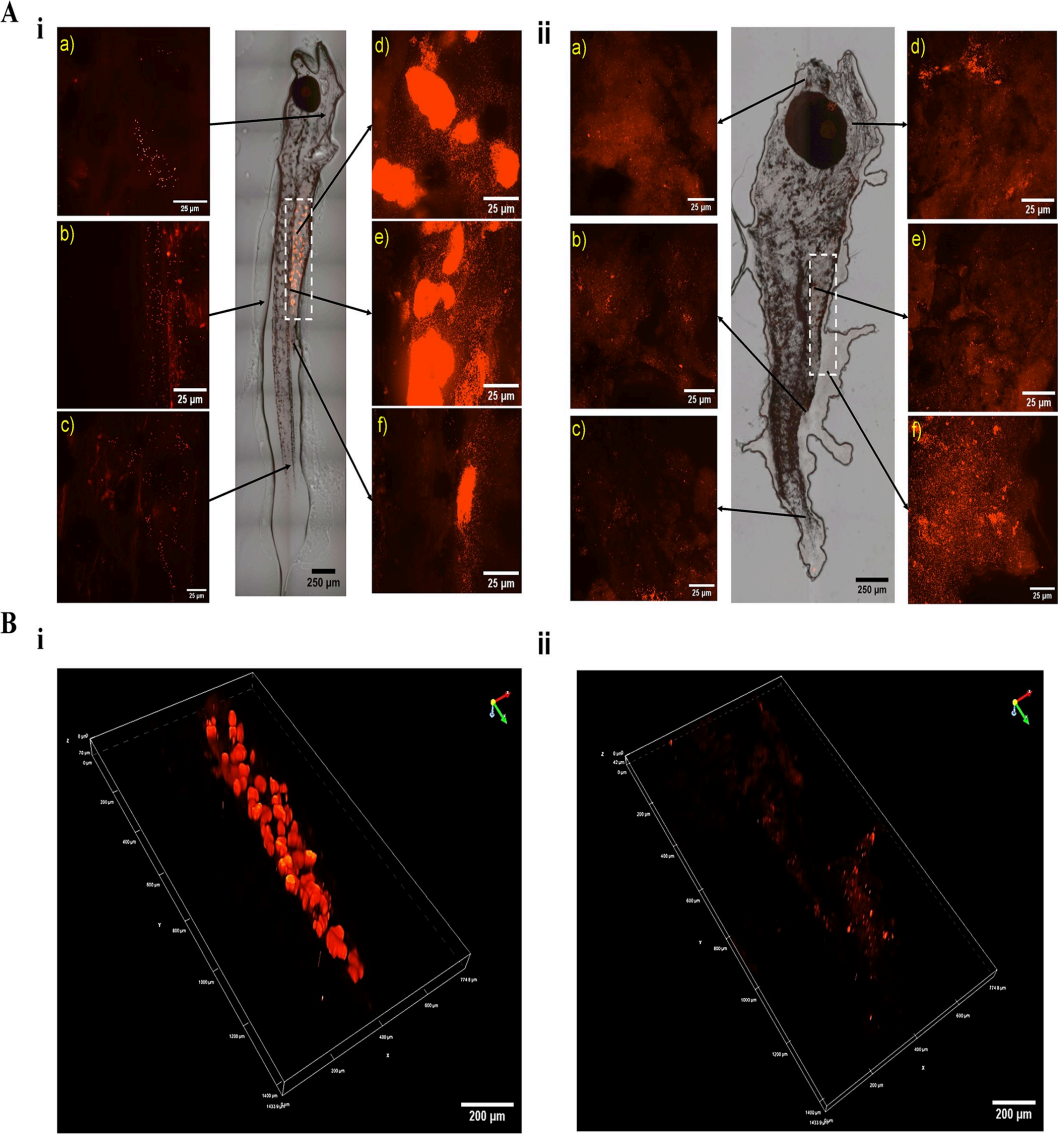
Fluorescent and bright-field images showing the biodistribution of NPs in *C*. *hippurus* after trophic transfer and depuration. A) Superimposed fluorescent and bright-field images of *C*. *hippurus* after (i) 48 hours of NP exposure via dietary intake, followed by (ii) a 72-hour depuration period. B) 3D z-stacked fluorescent images of the gut area indicated by the white dotted line in Fig 5A (i & ii).

Uptake and retention of NPs in *C*. *hippurus* following various durations of exposure and depuration were observed as well as transfer of these NPs to different organs or body parts. Fig 5 shows superimposed bright field and fluorescence images [Fig 5A(i-ii)], and 3D z-stacked fluorescence images [Fig 5B(i-ii), focused on the gut area indicated by the white dotted line in Fig 5A] of *C*. *hippurus* exposed to NPs via dietary intake for 48 h [Fig 5A(i)], followed by a 72-h depuration period [Fig 5A(ii)]. These conditions were selected as model samples because they represent the highest NP ingestion level and the longest subsequent depuration, as described in sections 3.1 and 3.2, respectively. The images show the highest concentration of NPs in the gut area after exposure [Fig 5A(i)], with 3D fluorescence images [Fig 5A(ii)] illustrating NPs predominantly clustered in distinct lumps. Close inspection reveals a significant quantity of NPs dispersed from these clusters and distributed throughout other parts of the gut [Fig 5A(i-d&e)]. Even after 72 h of depuration, during which nearly 95% of NPs were expelled (Fig 4B), a substantial quantity of NPs remained in the gut [Fig 5A(ii-e) & 5B]. Additionally, fluorescence images captured NPs around the anus area both after exposure and depuration [Fig 5A-i(f) & ii(b)]. NPs were also visible in other body regions, including areas near major organs such as the heart, liver, and gall bladder [Fig 5A-i(a) & ii(d)], as well as the head [Fig 5A(ii-a)], caudal peduncle [Fig 5A-i(c) & ii(c)], and fin areas [Fig 5A(i-b) & (ii-f)].

### 3.4 Effect of nanoplastics on growth

**Fig 6:**
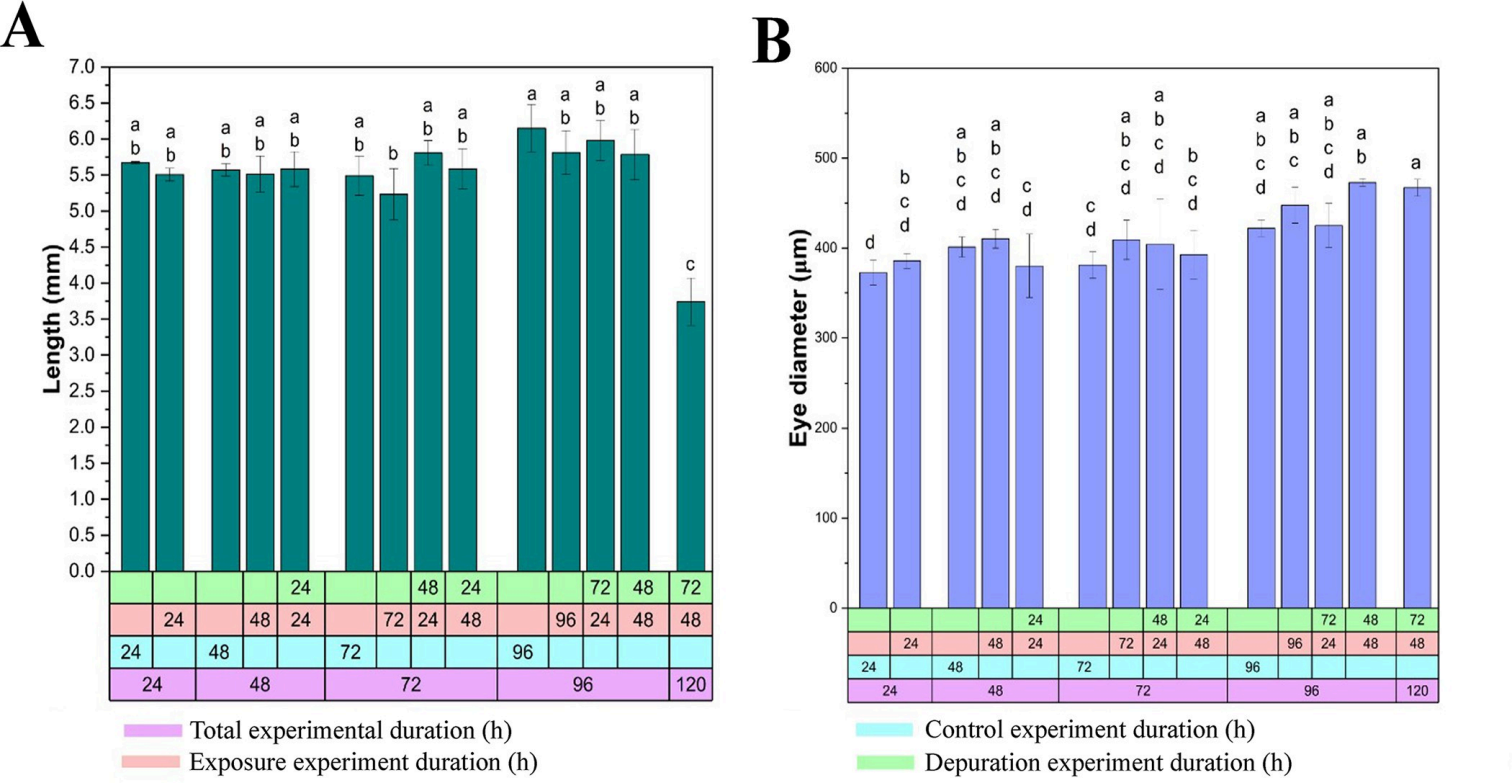
Effects of trophic transfer of NPs on the growth of *C*. *hippurus* larvae. (A) Length and (B) eye diameter measurements after different exposure durations and subsequent depuration periods.

Fig 6 shows the effects of NP exposure and depuration on the body length and eye diameter of *C*. *hippurus* larvae. To account for age-related variations, larvae of the same age were grouped, and the total experimental duration was divided into control, exposure, and/or depuration phases. In this study, NP exposure through trophic transfer, along with subsequent depuration, did not significantly affect the body length of *C*. *hippurus* larvae under any experimental conditions when compared to control groups of the same age (Fig 6A). Notably, only the larvae in the 120-h experimental condition (48 h of exposure followed by 72 h of depuration) showed significantly shorter lengths compared to other groups. Additionally, no significant differences in eye diameter were found between same-age groups (Fig 6B).

### 3.5 Histopathological change

**Fig 7:**
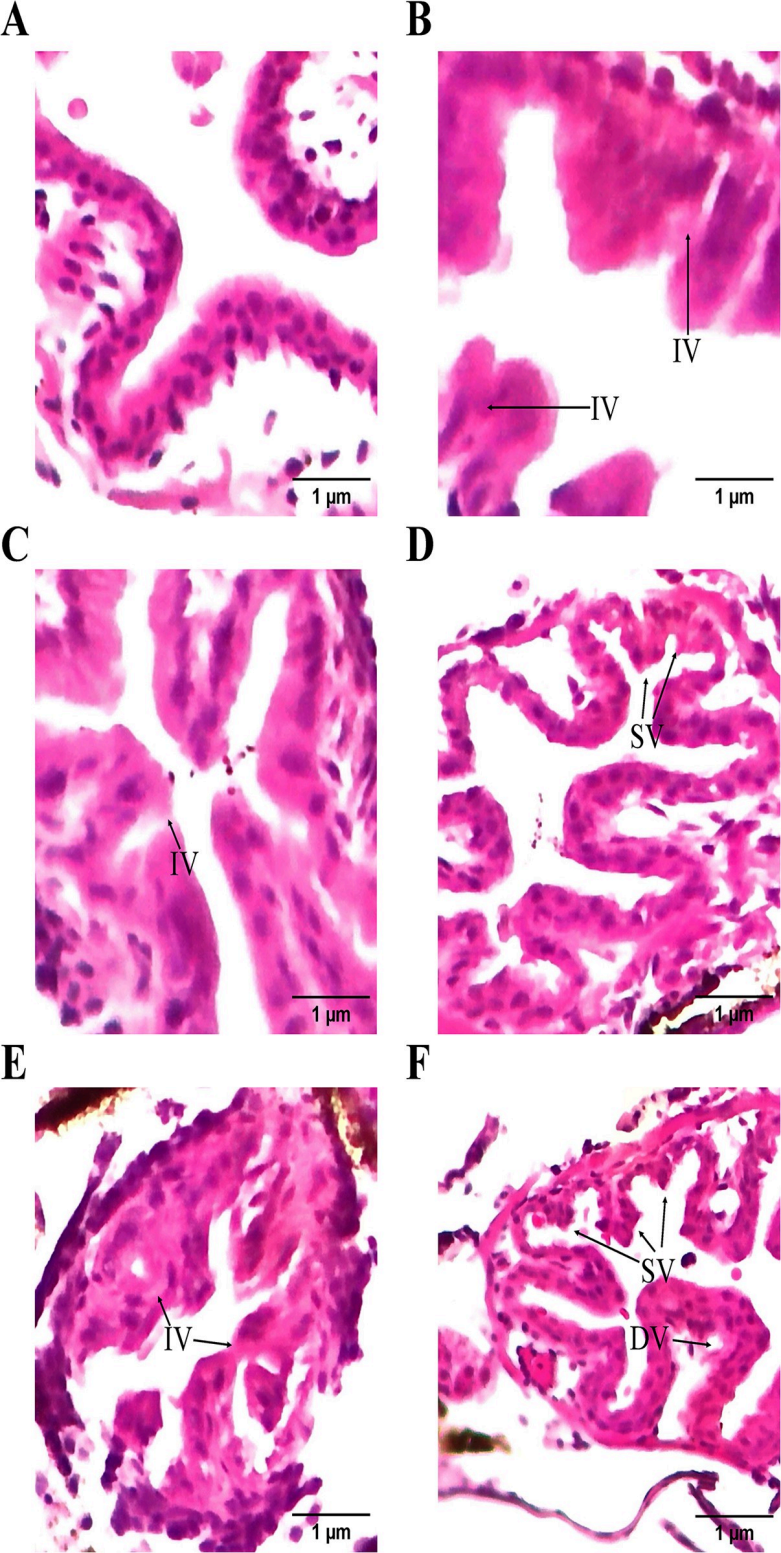
Histopathological changes in the intestine of *C*. *hippurus larvae* following exposure to NPs via trophic transfer and subsequent depuration. H&E-stained intestine sections of: (A) Control group, and larvae exposed to nanoplastics for (B) 24 h, (C) 48 h, (D) 72 h, (E) 96 h, and (F) 24 h of exposure followed by 72 h of depuration. Integration of villi (IV), shortening of villi (SV), and degradation of villi (DV).

Fig 7 presents the histopathological changes in the H&E-stained intestine of *C*. *hippurus* larvae after exposure to NPs and subsequent depuration. The images [Fig 7B–7E] show intestinal injury marked by shortening, integration, and degradation of villi in NP-exposed larvae, in contrast to the control group (Fig 7A). Even after 72 h of depuration following 24 h of exposure, similar intestinal changes were evident (Fig 7F).

## 4. Discussion

Our findings reveal that *C*. *hippurus* larvae ingested substantial quantities of NPs through trophic transfer, with uptake dynamics varying over time. Initially, high ingestion of NPE *B*. *plicatilis* and minimal egestion led to significant bioaccumulation while over time, increased NP egestion and reduced ingestion corresponded to fluctuations in the FI, suggesting alterations in feeding behavior. As *C*. *hippurus* adjusts feeding based on energetic satiation [70], disrupted feeding patterns suggest PS NPs may affect digestive enzyme activities, such as increased levels of lipase, chymotrypsin, and trypsin (possibly due to futile digestion attempts or NP-induced starvation) [36] and decreased amylase levels (potentially due to suppressed carbohydrate metabolism) [36,71]. This could even be exacerbated by the alteration of gut microbiota diversity, potentially leading to dysbiosis, as well as increased levels of cytokines such as *IL-1β*, *IL-8*, *IL-10*, and *TNF-α*, indicating intestinal inflammation, as seen in Zebrafish (*Danio rerio*) exposed to PS NPs [72].

NP excretion occurs mainly through feces [73]. Although a depuration period effectively reduced bioaccumulation, consistent with prior studies [71,74,75], significant retention of NPs was observed. One key factor in retention is the potential for re-exposure during depuration—possibly due to NP breakdown in feces and subsequent waterborne redistribution. Additionally, non-NPE *B*. *plicatilis* might ingest excreted NPs and be consumed again by *C*. *hippurus*, acting as NP vectors within the food web. This phenomenon highlights the need for caution in trophic transfer experiments and analysis while also raising concerns about the persistent nature of NP contamination in marine ecosystems.

The biodistribution of NPs within the larvae showed the highest concentration in the gut area, aligning with dietary ingestion as the primary route of NP entry in this study. Potential interactions of NPs with mucus secretions in the gut can trap and immobilize particles, leading to prolonged retention [73]. This finding aligns with previous studies of trophic transfer of PS NPs to Zebrafish via *P*. *caudatum* [44] and *Zacco temminckii* via multiple trophic levels [76]. NP size [77] and aggregation in seawater likely influence retention [27], as larger or clustered NPs may be excreted more slowly [75]. Additionally, NPs may cross biological barriers, such as the intestinal endothelium [78], due to increased permeability linked to cadherin protein upregulation [79], which could facilitate NP persistence and possible translocation to tissues beyond the gut. Evidence of NP presence in areas housing vital organs suggests they might traverse inter-tissue gaps [77] or via vasculature [80,81], raising concerns about systemic distribution. Furthermore, the presence of NPs in the fin areas may result from NP excretion by the larvae, followed by subsequent waterborne contact or internal transit within the fish. Similar studies have shown NP translocation to gills, brain, and muscle in *Macquaria novemaculeata* [82], and skin, muscle, gills, and liver in *Aphaniops hormuzensis* [83] exposed through the food chain, indicating that trophic transfer may lead to similar biodistribution patterns across species. Unlike previous studies that used organ dissections for precise NP accumulation measurement [81,82,84], the whole-larvae fluorescence imaging approach used here limited the resolution of localization within individual body parts. Instead, we inferred approximate physiological positions based on the observed fluorescence patterns.

Previous studies have examined fish length and eye diameter to assess the impacts of NPs on growth and potential morphological abnormalities [36,85]. These metrics provide insight into how NPs might affect developmental processes, with some evidence suggesting that NPs may reduce growth rate by limiting food intake [86] or restrict eye diameter by decreasing retinal cell numbers through apoptosis—a mechanism observed with other stressors, such as certain food colorants [87]. Interestingly, in our study, PS NP exposure through trophic transfer, with or without subsequent depuration, did not significantly impact the length or eye diameter of *C*. *hippurus* larvae under most conditions, which may relate to the relatively short experimental duration (maximum 96 h of exposure). In fact, only one experimental condition (48-h exposure followed by 72-h depuration), showed shorter lengths in larvae, though similar exposure duration without extended depuration did not yield this result, suggesting the effect may be unrelated to NP exposure or potentially a delayed impact. These observations align with findings in the literature, reporting NP and MP effects on growth and eye diameter in some fish and other aquatic organisms [86,88,89], while others report no significant impact [36,85,90]. Such variation may stem from differences in species, NP characteristics, and experimental conditions [85], highlighting the complexity of assessing NP impacts across diverse biological systems.

NP accumulation in the gut area suggested potential tissue damage in the intestine, prompting a closer look at histopathological changes. Histological analysis revealed intestinal injuries in *C*. *hippurus* larvae exposed to NPs, including villi shortening, integration and degradation, with similar effects persisting after depuration. This may explain why food uptake dropped significantly with increasing exposure duration. Such tissue degradation likely reflects a defensive response, where villi integration minimizes the contact area between tissue and pollutants [91]. This type of damage aligns with findings in other fish species exposed to PS NPs, including largemouth bass (*Micropterus salmoides*) [92], zebrafish [93], and grass carp (*Ctenopharyngodon idella*) [94]. Tissue damage from NP exposure is commonly linked to reactive oxygen species (ROS) generation and oxidative stress [95], resulting from limited antioxidant production, such as catalase (CAT) and superoxide dismutase (SOD) [96]. Although these antioxidants serve as defense mechanisms, excessive levels can harm critical biomolecules, including DNA and proteins [91,97], underscoring the complexity of NP-induced oxidative damage in aquatic organisms.

## 5. Conclusion

In this study, we investigated the transfer of polystyrene NPs to the commercially and ecologically significant fish species *Coryphaena hippurus* (mahi-mahi) via a two-step food chain involving *Brachionus plicatilis* (rotifers). We also examined the effects of depuration on *C*. *hippurus* when exposed to NP-free *B*. *plicatilis*. Our findings revealed that NP uptake initially increased significantly with time but later decreased. While depuration effectively reduced NP levels, a considerable portion of NPs persisted, primarily within the gut, underscoring potential health risks for fish and the possibility of NP trophic transfer to humans. Occasional increases in NP uptake during depuration may suggest re-exposure due to fecal NP release from other larvae. Biodistribution analysis demonstrated that most NPs accumulated in the gut, forming clusters, with smaller amounts translocating to regions housing vital organs like the heart and liver, as well as the head and caudal peduncle. Despite no significant effects on body length or eye diameter over the study period, histopathological analysis revealed considerable gut damage—including villi integration, shortening, and degradation—irrespective of exposure duration or depuration, suggesting potential impacts on long-term nutrient absorption and growth.

This study underscores the impacts of NP uptake via the foodborne route in *C*. *hippurus* larvae, revealing insights into bioaccumulation, biodistribution, growth effects, and intestinal tissue damage. Exploring a wider range of NP sizes and concentrations could provide a more comprehensive understanding of how these variables influence observed outcomes. Additionally, incorporating real-time monitoring could enhance the assessment of dynamic NP interactions over time. Future studies could benefit from microfluidic technologies, which allow for fish larvae [98–100] and NP exposure [15] studies with the possibility of continuous monitoring [15,101]. Microfluidic integrated sensors [102,103], actuators [104–106] as well as image processing of moving animals using artificial intelligence [107,108], can provide more detailed information on NP impacts and facilitate experimental automation. While fluorescence microscopy effectively highlighted NP biodistribution, complementing this approach with additional techniques could improve quantification within specific organs and tissues, offering a deeper insight into NP biodistribution at the micro-scale.

The significance of this work lies in its foundational insights into NP uptake and retention through trophic transfer in a key marine species at a vulnerable life stage. These findings contribute to a growing body of evidence on the ecological risks associated with NP contamination in marine environments. Persistent NP retention and tissue damage could have profound effects on fish health and population sustainability, with implications that extend to ecosystem stability and human exposure through the food web. By establishing groundwork in trophic transfer and NP biodistribution, this study informs future research pathways critical to understanding and mitigating the impacts of NPs across marine food chains.

## Supporting information

S1 Graphical abstract (TIF)
