# Supplementary figures and images for: Trophic transfer and bioaccumulation of nanoplastics in Coryphaena hippurus (mahi-mahi) and effect of depuration
Source: PLoS One. 2024 Nov 21;19(11):e0314191. doi: 10.1371/journal.pone.0314191 (PMC11581304; doi:10.1371/journal.pone.0314191)

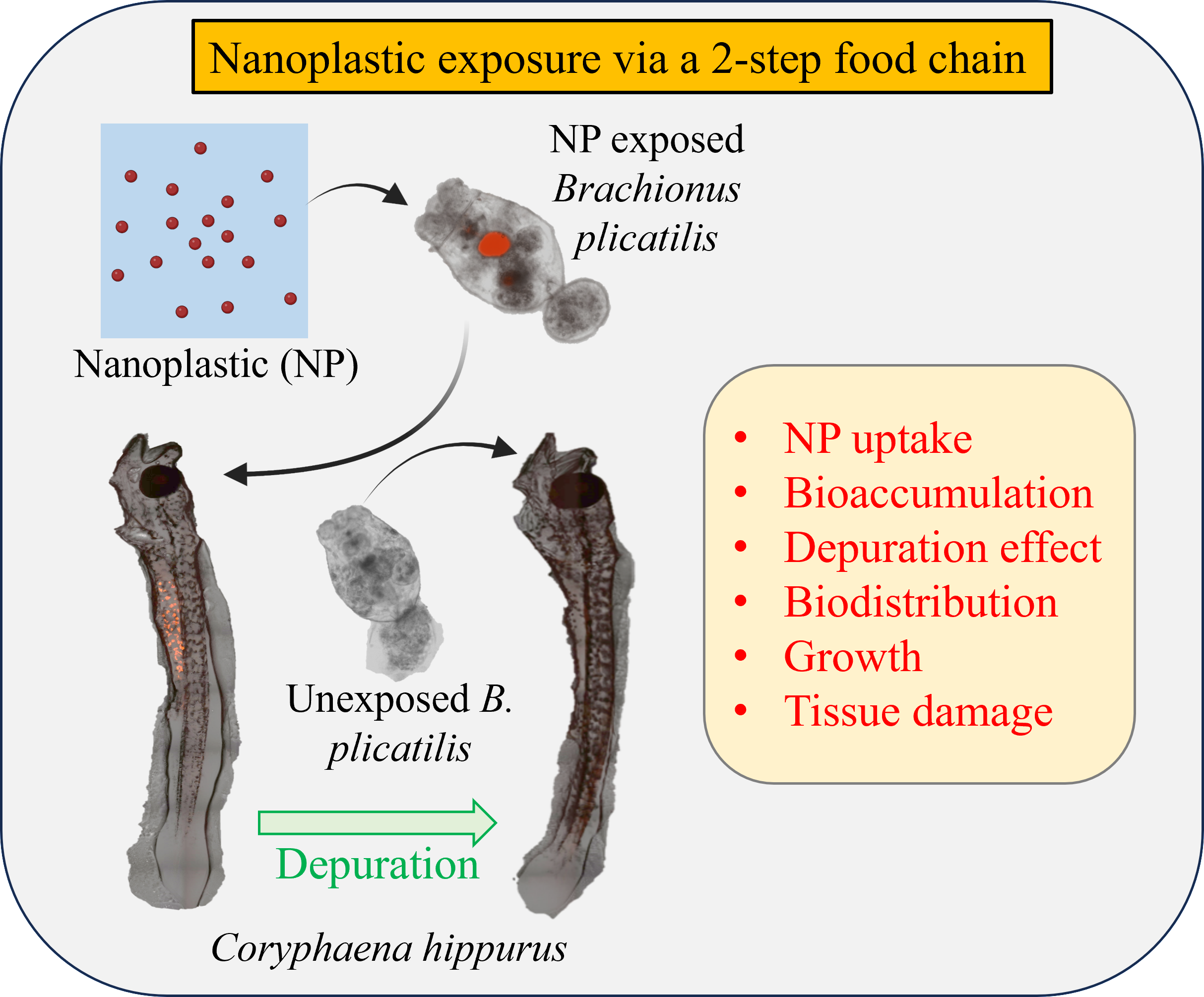

Supplement: S1 Graphical abstract — (TIF) [file pone.0314191.s001.tif]
